# Fine Tuning of Cholinesterase and Glutathione-S-Transferase Activities by Organoruthenium(II) Complexes

**DOI:** 10.3390/biomedicines9091243

**Published:** 2021-09-16

**Authors:** Tomaž Trobec, Kristina Sepčić, Monika Cecilija Žužek, Jerneja Kladnik, Nina Podjed, Catarina Cardoso Páscoa, Iztok Turel, Robert Frangež

**Affiliations:** 1 Institute of Preclinical Sciences, Veterinary Faculty, University of Ljubljana, 1000 Ljubljana, Slovenia; tomaz.trobec@vf.uni-lj.si (T.T.); monika.zuzek@vf.uni-lj.si (M.C.Ž.); 2 Department of Biology, Biotechnical Faculty, University of Ljubljana, 1000 Ljubljana, Slovenia; 3 Department of Chemistry and Biochemistry, Faculty of Chemistry and Chemical Technology, University of Ljubljana, 1000 Ljubljana, Slovenia; jerneja.kladnik@fkkt.uni-lj.si (J.K.); nina.podjed@fkkt.uni-lj.si (N.P.); c.pascoa@campus.fct.unl.pt (C.C.P.); 4 NOVA School of Science and Technology, Universidade Nova de Lisboa, Campus de Caparica, 2829-516 Caparica, Portugal

**Keywords:** cholinesterase, enzyme inhibition, glutathione S-transferase, organoruthenium complex, pyrithione, *β*-diketone, carbonyl

## Abstract

Cholinesterases (ChEs) show increased activities in patients with Alzheimer’s disease, and remain one of the main therapeutic targets for treatment of this neurodegenerative disorder. A library of organoruthenium(II) complexes was prepared to investigate the influence of their structural elements on inhibition of ChEs, and on another pharmacologically important group of enzymes, glutathione S-transferases (GSTs). Two groups of organoruthenium(II) compounds were considered: (i) organoruthenium(II) complexes with *p*-cymene as an arene ligand, and (ii) organoruthenium(II) carbonyl complexes as CO-releasing molecules. Eight organoruthenium complexes were screened for inhibitory activities against ChEs and GSTs of human and animal origins. Some compounds inhibited all of these enzymes at low micromolar concentrations, while others selectively inhibited either ChEs or GSTs. This study demonstrates the importance of the different structural elements of organoruthenium complexes for their inhibitory activities against ChEs and GSTs, and also proposes some interesting compounds for further preclinical testing as ChE or GST inhibitory drugs.

## 1. Introduction

For at least 3500 years, precious metals have been used for different medicinal purposes, and it is now known that the medicinal properties of metals are linked to their specific biological effects. As many metal ions (e.g., zinc, copper, iron) are involved in several physiological processes, there is great scope for designing metal-based therapeutic agents [1]. Factors that have critical influences on the biological activities of metal complexes include the nature and oxidation state of the metal ion, the number and types of bonded ligands and the coordination geometry [2,3,4].

Ruthenium compounds show a broad spectrum of biological activities, which range from immunosuppressant, antibacterial, antiviral and antitumour, to antiparasitic effects. These activities are due to selective inhibition of many medicinally essential enzymes that are involved in different pathological conditions. These enzymes comprise in particular the cholinesterases (ChEs), glutathione S-transferases (GSTs), protein kinases, aldo-keto reductase, thioredoxin reductase, cathepsin B, topoisomerase II and HIV−1 reverse transcriptase, along with many others [5,6,7,8,9,10,11]. Over the last decade, we have focused on the study of the inhibitory activities of different organoruthenium(II) compounds against various enzymes, including ChEs (i.e., acetylcholinesterases (AChEs) and butyrylcholinesterases (BChEs)) and GSTs [12,13]. In humans and other animals, the altered functions of these enzymes result in different pathological conditions (e.g., Alzheimer’s disease; breast, ovarian, colorectal, pancreatic cancers). From this point of view, the ChEs and GSTs are critical therapeutic targets.

Acetylcholinesterase is a specific serine hydrolase that predominantly catalyses the hydrolysis of the neurotransmitter acetylcholine. AChE is present in synapses of the central and peripheral nervous systems, in both motor and sensory nerve fibres. It is also found in neuromuscular junctions, cholinergic synapses of the autonomic nervous system, and in erythrocytes [14,15]. BChE is a non-specific ChE that hydrolyses a wide variety of choline and non-choline esters, and is present throughout the body. In the brain, BChE is associated with glial and endothelial cells [16]. AChE and BChE are involved in the pathogenesis of diseases, such as Alzheimer’s disease and type II diabetes mellitus [17].

Alzheimer’s disease is a chronic multifactorial neurodegenerative disease [18] in which ChEs are among the most important therapeutic targets. Alzheimer’s disease is a result of several abnormalities, which include decreased levels of acetylcholine in the brain, amyloid-β protein aggregation, protein tau hyperphosphorylation, altered metal levels, oxidative stress, dysfunction or loss of cholinergic neurons, and reactive gliosis [19,20,21]. The function of ChEs in patients with Alzheimer’s disease is altered. In the early stages of the disease, the expression of AChE is enhanced. With progression of the disease, the AChE expression decreases to only 33% to 45% of its normal levels, while the expression of BChE is enhanced by as much as 40% to 90% of the normal values in specific brain areas [17]. Consequently, patients with Alzheimer’s disease suffer from acetylcholine deficit, which is reflected in a loss of cognitive functions.

AChE also has a crucial role in the amyloid-β protein aggregation process, in which a stable AChE−amyloid-β complex is formed, which then accelerates amyloid-β protein aggregation and the formation of amyloid plaques [21]. Current symptomatic treatment of Alzheimer’s disease is aimed at enhancing the acetylcholine levels with the use of ChE inhibitors that prolong the lifetime of acetylcholine in synapses and improve cognitive functions for patients with Alzheimer’s disease (e.g., tacrine, galantamine, donepezil, rivastigmine) [21,22]. This cholinergic strategy after all of these years remains a promising approach for Alzheimer’s disease drug development. On this basis, it remains crucial to develop compounds that can inhibit both AChE and BChE [23]. For this, the organoruthenium(II) compounds are very promising due to their inhibitory properties against both AChE and BChE.

The GSTs represent another group of enzymes that might serve as attractive molecular targets for the organoruthenium(II) compounds. GSTs belong to the family of phase II detoxification enzymes [24]. They can catalyse glutathione conjugation to a wide variety of endogenous and exogenous electrophilic compounds, which yields more water-soluble products and facilitates their elimination [24,25,26]. These processes protect different macromolecules from attack by reactive electrophiles, including environmental carcinogens, reactive oxygen species and chemotherapeutic agents [27]. GSTs are divided into three major groups according to their cellular localisation: cytosolic, mitochondrial and microsomal.

As well as being involved in detoxification, GSTs have many other biological functions, which include protection of cells against oxidative stress, involvement in synthesis and modification of leukotrienes and prostaglandins, and modulatory effects on the signal transduction pathways for cell survival and apoptosis [25,26]. GSTs also have essential roles in the development of resistance to anticancer drugs, which is a key element in the failure of chemotherapy. Cancer cells often show high GST expression compared to normal cells [25]. In a wide variety of human cancers (e.g., breast, ovarian, colorectal, pancreas, and many others), the overexpression and enhanced activities of GSTs can contribute to increased detoxification of anticancer drugs [25,26]. It also appears that GSTs can interact with efflux transporters to increase efflux of chemotherapeutics from cells [26]. Both of these processes can result in development of resistance to chemotherapeutics. This indicates the importance of using GST inhibitors against certain types of cancers.

**Figure 1:**
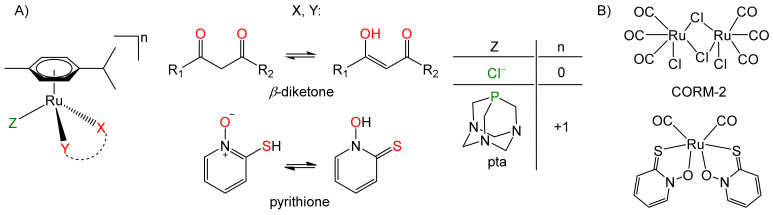
(**A**) *p*-Cymene-containing organoruthenium(II) complexes; (**B**) Organoruthenium(II) carbonyl complexes as CO-releasing molecules (CORM−2; i.e., [Ru(CO)_3_Cl_2_]_2_). Atoms labelled in red and green represent the donor atoms through which the ligands are bound to the ruthenium ion.

**Figure 2:**
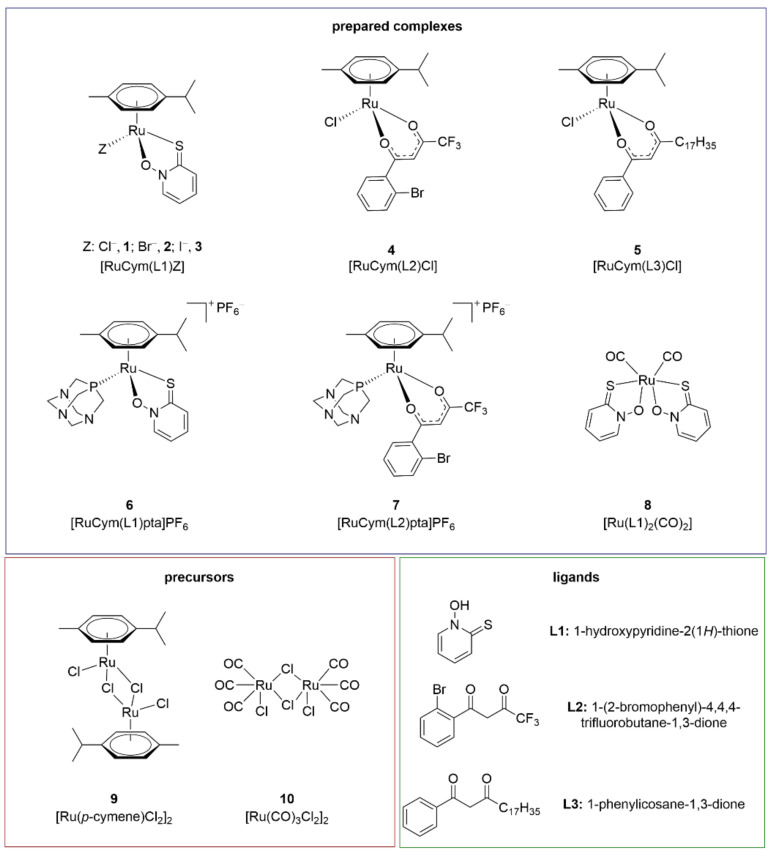
Structures of the investigated complexes (**1**–**8**), precursors (**9**–**10**) and ligands (**L1**–**L3**). The syntheses of complexes **1** and **6** were previously reported in [8,33].

In the present study, we focused on anti-ChE and anti-GST activities of two groups of ruthenium(II) compounds: organoruthenium(II) complexes with *p*-cymene as an arene ligand (Figure 1A), and organoruthenium(II) carbonyl complexes as CO-releasing molecules (CORMs) (Figure 1B). Organoruthenium(II) complexes with *β*-diketonates and chlorido/pta ligands have been well investigated and are known for their biological activities, and especially their anticancer effects [28,29,30,31]. In recent years, we have also focused on the biological properties of organoruthenium(II) complexes with the ligand pyrithione (1-hydroxypyridine-2(1*H*)-thione, 2-mercaptopyridine *N*-oxide) and analogues, and we have shown their encouraging anticancer activities [8,32,33], as well as their antineurodegenerative properties [12,34]. Recently, organoruthenium(II) complexes with arene ligands have received a lot of attention. However, nowadays, CORM-type compounds are also becoming more and more attractive due to their CO-releasing roles, as at adequate concentrations and under specific ways of application, CO has shown cardioprotective, anti-inflammatory, antiproliferative and proapoptotic properties [35,36]. Therefore, these organoruthenium(II) complexes were tested here for anti-ChE and anti-GST activities. Figure 2 illustrates the structures of all ligands and complexes used in this study.

## 2. Materials and Methods

### 2.1. General

All of the reagents were from commercial sources and were used as received. The precursors dichloro(*p*-cymene) ruthenium(II) dimer [Ru(*p*-cymene)Cl_2_]_2_ (**9**) and tricarbonyldichlororuthenium(II) dimer [Ru(CO)_3_Cl_2_]_2_ (CORM-2, **10**) were from Strem Chemicals, and ligands **L1**–**L3** were from Fluorochem; 1,3,5-triaza-7-phosphaadamantane (pta) was prepared as reported previously [37]. Thin layer chromatography (TLC) was carried out with pre-coated TLC sheets (Alugram SIL G/UV254; Macherey-Nagel, Düren, Germany). Column chromatography was carried out using silica gel 60 (35–70 mm; Merck, Darmstadt, Germany) or aluminium oxide (Riedel-de Haën) as stationary phases.

^1^H nuclear magnetic resonance (NMR) spectra were obtained at 500 MHz (Avance III 500; Bruker BioSpin GmbH, Rheinstetten, Germany). The data were processed using the MestReNova programme version 11.0.4 [38]. Chemical shifts (δ) are given in ppm and coupling constants (*J*) in Hz. Multiplicity is defined as s = singlet, d = doublet, dd = doublet of doublets, t = triplet, td = triplet of doublets, sept = septet and m = multiplet. Chemical shifts of the ^1^H NMR spectra are referenced to residual peaks of CDCl_3_, acetone-*d_6_* and dimethylsulphoxide-*d_6_* (DMSO-*d_6_*) at 7.26, 2.05 and 2.50 ppm, respectively. Infrared (IR) spectra were recorded in the range from 4000 cm^–1^ to 600 cm^–1^ (Spectrum 100; Perkin-Elmer, Shelton, CT, USA), or in the range from 4000 cm^–1^ to 400 cm^–1^ (Alpha II FT-IR; Bruker, Billerica, MA, USA). The attenuated total reflection (ATR) module was used on both instruments. Elemental analysis was performed for carbon, hydrogen and nitrogen (2400 II analyser; Perkin-Elmer, Waltham, MA, USA). Electrospray ionization high-resolution mass spectra (ESI-HRMS; 6224 Accurate Mass TOF LC Mass Spectrometer; Agilent Technologies, Santa Clara, CA, USA) and ultraviolet-visible spectra (UV-Vis; Lambda 750 UV/Vis/NIR; Perkin-Elmer, Waltham, MA, USA) were recorded. Single crystal X-ray diffraction data for compounds [RuCym(L2)Cl] (**4**) and [Ru(L1)_2_(CO)_2_] (**8**) were collected on a diffractometer (SuperNova; Agilent Technologies XRD Products, Oxfordshire, UK) with a molybdenum (Mo-K*_α_*, *λ* = 0.71073 Å) micro-focus sealed X-ray source at 150 K. The diffractometer was equipped with mirror optics and an Atlas detector. CrysAlis PRO [39] was used for data processing. Structures were solved with the Olex^2^ software [40] using ShelXT [41], and refined using least squares methods in ShelXL [42]. Anisotropic displacement parameters were determined for all non-hydrogen atoms. Hydrogen atoms were placed in the geometrically calculated positions and refined using riding models. The Platon [43] and Mercury [44] programmes were used for crystal structure analysis and preparation of figures. Both crystal structures have been deposited with the Cambridge Crystallographic Data Centre, and were assigned the deposition numbers 2067254 (complex **4**) and 2067253 (complex **8**).

### 2.2. Synthesis

[RuCym(L1)Cl] (**1**). Complex **1** was synthesised as reported previously [33].

[RuCym(L1)Br] (**2**). For the preparation of complex **2**, complex **1** was initially prepared according to the procedure reported previously [33]. Then, a mixture of complex **1** (0.202 mmol, 1 equiv.) and AgNO_3_ (0.524 mmol, 2.6 equiv.) was stirred in MeOH at room temperature in the dark for 1 h. The precipitated AgCl was filtered off through fine Celite powder. KBr (3.361 mmol, 16.7 equiv.) was added to the filtrate and the mixture was stirred further at room temperature in the dark for 45 min. The solvent was then evaporated and dichloromethane was added, resulting in the precipitation of KNO_3_, which was filtered off through fine Celite powder. The filtrate was concentrated on a rotary evaporator to around 2 mL. The complex was precipitated by addition of *n*-heptane, with the dark red-brown solid filtered off and dried at 45 °C. Yield: 44 mg, 49%. ^1^H NMR (500 MHz, CDCl_3_): δ 8.01 (dd, 1H, *J* = 6.8, 0.7 Hz, Ar–*H* L1), 7.43 (dd, 1H, *J* = 8.3, 1.3 Hz, Ar–*H* L1), 7.06–6.99 (m, 1H, Ar–*H* L1), 6.71 (td, 1H, *J* = 6.8, 1.7 Hz, Ar–*H* L1), 5.49 (d, 2H, *J* = 6.0 Hz, Ar–*H* cym), 5.27 (d, 2H, *J* = 6.0 Hz, Ar–*H* cym), 2.86 (sept, 1H, *J* = 6.9 Hz, Ar–C*H*(CH_3_)_2_ cym), 2.27 (s, 3H, Ar–C*H*_3_ cym), 1.28 (d, 6H, *J* = 6.9 Hz, Ar–CH(C*H*_3_)_2_ cym) ppm. IR (ATR, cm^–1^, selected bands): 2963, 2871, 1845, 1594, 1547, 1455, 1236, 1134, 757. Elemental analysis calcd. for C_15_H_18_BrNORuS (%): C, 40.82; H, 4.11; N, 3.17. Found (%): C, 40.74; H, 3.89; N, 3.11. UV-Vis (λ [nm], ε [L mol^–1^ cm^–1^] at c = 5.0 ×10^–5^ mol L^–1^, MeOH): 284 (10660), 359sh (1845), 486 (487). ESI-HRMS *m/z* calcd. for [M–Br]^+^: 362.0153, found: 362.0146.

Diiodo(*p*-cymene)ruthenium(II) dimer; [Ru(*p*-cymene)I_2_]_2_. For the preparation of the complex **3**, [Ru(*p*-cymene)I_2_]_2_ was initially prepared. A mixture of ruthenium precursor **9** (0.262 mmol, 1 equiv.) and AgNO_3_ (2.070 mmol, 7.9 equiv.) was stirred in MeOH at room temperature in the dark for 1 h. Then, KI (3.352 mmol, 12.8 equiv.) was added and the mixture was left to stir for an additional 15 min. The solvent was then evaporated, and the crude product dissolved in dichloromethane. The by-product salts that precipitated were filtered off through fine Celite powder. The filtrate was concentrated to around 2 mL, and after addition of hexane, the complex precipitated, and was filtered off and dried at 45 °C. ^1^H NMR (500 MHz, CDCl_3_): 5.53 (d, 4H, *J* = 5.9 Hz, Ar–*H* cym), 5.43 (d, 4H, *J* = 5.9 Hz, Ar–*H* cym), 3.01 (sept, 2H, *J* = 6.9 Hz, Ar–C*H*(CH_3_)_2_ cym), 2.36 (s, 6H, Ar–C*H*_3_ cym), 1.25 (d, 12H, *J* = 6.9 Hz, Ar–CH(C*H*_3_)_2_ cym) ppm.

[RuCym(L1)I] (**3**). A mixture of [Ru(*p*-cymene)I_2_]_2_ (0.083 mmol, 1 equiv.), ligand 1-hydroxypyridine-2(1*H*)-thione (**L1**; 0.246 mmol, 3 equiv.) and the base NaOMe (0.261, 3.1 equiv.) was stirred in acetone at room temperature overnight. The next day, the solvent was evaporated, and the crude product was purified by column chromatography using silica gel as stationary phase (mobile phase, 5% acetone in dichloromethane). After combining the appropriate fractions, the mobile phase was removed on a rotary evaporator, and the dark red-brown solid was precipitated from a dichloromethane/*n*-heptane solvent combination, and filtered off and dried at 45 °C. Yield: 20 mg, 25%. ^1^H NMR (500 MHz, CDCl_3_): δ 7.96 (dd, 1H, *J* = 6.8, 0.7 Hz, Ar–*H* L1), 7.40 (dd, 1H, *J* = 8.3, 1.5 Hz, Ar–*H* L1), 7.04–7.00 (m, 1H, Ar–*H* L1), 6.71 (td, 1H, *J* = 6.8, 1.7 Hz, Ar–*H* L1), 5.54 (d, 2H, *J* = 6.0 Hz, Ar–*H* cym), 5.28 (d, 2H, *J* = 6.0 Hz, Ar–*H* cym), 2.90 (sept, 1H, *J* = 6.9 Hz, Ar–C*H*(CH_3_)_2_ cym), 2.28 (s, 3H, Ar–C*H*_3_ cym), 1.29 (d, 6H, *J* = 6.9 Hz, Ar–CH(C*H*_3_)_2_ cym) ppm. IR (ATR, cm^–1^, selected bands): 3486, 3429, 2960, 1607, 1547, 1455, 1234, 1133, 1087, 755. Elemental analysis calcd. for C_15_H_18_INORuS (%): C, 36.89; H, 3.72; N, 2.87. Found (%): C, 35.97; H, 3.39; N, 2.86. UV-Vis (λ [nm], ε [L mol^–1^ cm^–1^] at c = 5.0 ×10^–5^ mol L^–1^, MeOH): 284 (15117), 358sh (3084), 485 (846). ESI-HRMS *m/z* calcd. for [M–I]^+^: 362.0153, found: 362.0149.

[RuCym(L2)Cl] (**4**). The syntheses of ruthenium(II) chlorido complexes **4** and **5** followed a previously published, but slightly modified, procedure [45]. Ruthenium precursor **9** (0.163 mmol, 1 equiv.), *β*-diketonate ligand 1-(2-bromophenyl)-4,4,4-trifluorobutane-1,3-dione (**L2**; 0.346 mmol, 2.1 equiv.) and NaOMe base (0.359 mmol, 2.2 equiv.) were stirred in 10% MeOH in dichloromethane at room temperature overnight. The solvent was removed under reduced pressure, and the residue was dissolved in dichloromethane. The NaCl and other insoluble impurities precipitated in this solvent and were removed by filtration through fine Celite powder. The filtrate was concentrated on a rotary evaporator, and the product precipitated by addition of hexane. The orange solid was filtered off and dried at 45 °C, with no further purification required. Yield: 145 mg, 79%. ^1^H NMR (500 MHz, acetone-*d*_6_): δ 7.70–7.68 (m, 1H, Ar–*H* L2), 7.48–7.44 (m, 1H, Ar–*H* L2), 7.43–7.39 (m, 2H, Ar–*H* L2), 5.86 (d, 1H, *J* = 5.9 Hz, Ar–*H* cym), 5.81 (d, 1H, *J* = 5.9 Hz, Ar–*H* cym), 5.79 (s, 1H, CO–C*H*–CO L2), 5.54 (d, 1H, *J* = 5.9 Hz, Ar–*H* cym), 5.50 (d, 1H, *J* = 5.9 Hz, Ar–*H* cym), 2.93 (sept, 1H, *J* = 6.9 Hz, Ar–C*H*(CH_3_)_2_ cym), 2.24 (s, 3H, Ar–C*H*_3_ cym), 1.36 (2d, 6H, *J* = 6.9 Hz, Ar–CH(C*H*_3_)_2_ cym) ppm. IR (ATR, cm^–1^, selected bands): 2976, 1586, 1526, 1307, 1191, 1151, 1132, 1087, 1024, 777. Elemental analysis calcd. for C_20_H_19_BrClF_3_O_2_Ru (%): C, 42.53; H, 3.39. Found (%): C, 42.57; H, 2.94. UV-Vis (λ [nm], ε [L mol^–1^ cm^–1^] at c = 0.5 × 10^–4^ mol L^–1^, MeOH): 290 (11000), 361sh (3800). ESI-HRMS *m/z* calcd. for [M–Cl]^+^: 530.9544, found: 530.9551.

[RuCym(L3)Cl] (**5**). Ruthenium precursor **9** (0.163 mmol, 1 equiv.), *β*-diketonate ligand 1-phenylicosane-1,3-dione (**L3**; 0.357 mmol, 2.2 equiv.) and NaOMe (0.359 mmol, 2.2 equiv.) were stirred in 10% MeOH in dichloromethane at room temperature overnight. The solvent was removed under reduced pressure, and the residue was dissolved in dichloromethane. The NaCl and other insoluble impurities precipitated in this solvent and were removed by filtration through fine Celite powder. The filtrate was evaporated to dryness on a rotary evaporator. The crude orange product was purified by column chromatography using silica gel as stationary phase (mobile phases, dichloromethane, 10% MeOH in dichloromethane). The appropriate fractions were combined, the solvent was removed, and the orange product precipitated after drying at 45 °C. Yield: 68 mg, 32%. ^1^H NMR (500 MHz, CDCl_3_): δ 7.83–7.81 (m, 2H, Ar–*H* L3), 7.42–7.39 (m, 1H, Ar–*H* L3), 7.36–7.33 (m, 2H, Ar–*H* L3), 5.79 (s, 1H, CO–C*H*–CO L3), 5.53 (d, 1H, *J* = 5.8 Hz, Ar–*H* cym), 5.51 (d, 1H, *J* = 5.7 Hz, Ar–*H* cym), 5.25–5.22 (m, 2H, Ar–*H* cym), 2.95 (sept, 1H, *J* = 6.9 Hz, Ar–C*H*(CH_3_)_2_ cym), 2.40–2.29 (m, 2H, (C*H*_2_)_16_–CH_3_ L3, overlapped), 2.29 (s, 3H, Ar–C*H*_3_ cym, overlapped), 1.68–1.63 (m, 2H, (C*H*_2_)_16_–CH_3_ L3), 1.36 (2d, 6H, *J* = 6.9 Hz, Ar–CH(C*H*_3_)_2_ cym), 1.25 (m, 28H, (C*H*_2_)_16_–CH_3_ L3), 0.88 (t, 3H, *J* = 6.9 Hz, (CH_2_)_16_–C*H*_3_ L3) ppm. IR (ATR, cm^–1^, selected bands): 2918, 2849, 1588, 1548, 1519, 1490, 1391, 788, 712, 692. Elemental analysis calcd. for C_36_H_55_ClO_2_Ru (%): C, 65.88; H, 8.45. Found (%): C, 66.55; H, 8.72. UV-Vis (λ [nm], ε [L mol^–1^ cm^–1^] at c = 0.5 ×10^–4^ mol L^–1^, MeOH): 290 (16400), 368sh (4600). ESI-HRMS *m/z* calcd. for [M–Cl]^+^: 621.3246, found: 621.3247.

[RuCym(L1)pta]PF_6_ (**6**). Complex **6** was synthesised as reported previously [33].

[RuCym(L2)pta]PF_6_ (**7**). Ruthenium(II) pta complex **7** was synthesised according to a procedure published previously [30]. A mixture of ruthenium(II) chlorido complex **4** (0.177 mmol, 1 equiv.), pta (0.267 mmol, 1.5 equiv.) and silver salt AgPF_6_ (0.267 mmol, 1.5 equiv.) was stirred in acetone at room temperature for approximately 48 h in the dark. The solvent was removed under reduced pressure, and the residue was dissolved in dichloromethane. The insoluble AgCl salt that formed as a by-product of the reaction was removed by filtration through fine Celite powder. The filtrate was concentrated on a rotary evaporator, and the product was precipitated by addition of hexane. The crude product was filtered off and purified by column chromatography using silica gel as stationary phase (mobile phases, 5% MeOH in dichloromethane; 10% MeOH in dichloromethane). The appropriate fractions were combined and concentrated, and the product was precipitated with hexane, with the orange solid filtered off and dried at 45 °C. Yield: 53 mg, 36%. ^1^H NMR (500 MHz, DMSO-*d*_6_): δ 7.76 (dd, 1H, *J* = 7.6, 1.5 Hz, Ar–*H* L2), 7.68 (dd, 1H, *J* = 7.6, 1.5 Hz, Ar–*H* L2), 7.54–7.47 (m, 2H, Ar–*H* L2), 6.41 (d, 1H, *J* = 6.3 Hz, Ar–*H* cym), 6.29–6.25 (m, 3H, Ar–*H* cym), 6.20 (s, 1H, CO–C*H*–CO L2), 4.58–4.45 (m, 6H, *H* pta), 4.22 (s, 6H, *H* pta), 2.55 (sept, 1H, *J* = 7.0 Hz, Ar–C*H*(CH_3_)_2_ cym), 1.90 (s, 3H, Ar–C*H*_3_ cym), 1.17 (2d, 6H, *J* = 7.0 Hz, Ar–CH(C*H*_3_)_2_ cym) ppm. IR (ATR, cm^–1^, selected bands): 2969, 1584, 1308, 1199, 1147, 1013, 972, 946, 831, 730. Elemental analysis calcd. for C_26_H_31_BrF_9_N_3_O_2_P_2_Ru × 0.25 C_6_H_14_ (%): C, 38.72; H, 4.08; N, 4.93. Found (%): C, 38.64; H, 4.00; N, 4.75. Note that hexane was also seen in the ^1^H NMR spectra despite drying the sample on a vacuum line. UV-Vis (λ [nm], ε [L mol^–1^ cm^–1^] at c = 0.5 ×10^–4^ mol L^–1^, MeOH): 287 (9800), 368 (5200). ESI-HRMS *m/z* calcd. for [M–PF_6_]^+^: 688.0312, found: 688.0322.

[Ru(L1)_2_(CO)_2_] (**8**). Ruthenium precursor **10** (0.073 mmol, 1 equiv.), ligand **L1** (0.292 mmol, 4 equiv.) and the base NaOMe (0.292 mmol, 4 equiv.) were stirred in a mixture of MeOH and chloroform (3 mL, 5 mL, respectively) at room temperature for 30 min. Then, the solvents were evaporated, and dichloromethane was added. The NaCl that precipitated was filtered off through fine Celite powder. The filtrate was evaporated, and the crude product was purified by column chromatography using aluminium oxide (mobile phase, 2% acetone in dichloromethane). After combining the appropriate fractions, the mobile phase was removed on a rotary evaporator, and the pale-yellow solid that precipitated from the dichloromethane/*n*-heptane added was filtered off and dried at 45 °C. Yield: 37 mg, 62%. ^1^H NMR (500 MHz, acetone-*d*_6_): δ 8.17 (d, 2H, *J* = 6.9, Hz, Ar–*H* L1), 7.72 (dd, 2H, *J* = 8.4, 1.6 Hz, Ar–*H* L1), 7.45–7.40 (m, 2H, Ar–*H* L1), 7.08 (td, 2H, *J* = 6.9, 1.6 Hz, Ar–*H* L1) ppm. IR (ATR, cm^–1^, selected bands): 2041, 1957, 1545, 1455, 1408, 1237, 1133, 756, 746, 632. Elemental analysis calcd. for C_12_H_8_N_2_O_4_RuS_2_ (%): C, 35.21; H, 1.97; N, 6.84. Found (%): C, 34.97; H, 1.44; N, 6.72. UV-Vis (λ [nm], ε [L mol^–1^ cm^–1^] at c = 5.0 ×10^–5^ mol L^–1^, MeOH): 256 (23602), 290sh (12248), 340 (4111). ESI-HRMS *m/z* calcd. for [M + H]^+^: 410.9042, found: 410.9050.

### 2.3. Enzyme Inhibition Assays

#### 2.3.1. Cholinesterase Inhibition Assay

The activities of the ChEs were determined using a modification of the Ellman method [46] adapted for microtiter plates, as described in [47]. Stock solutions of complexes **4**, **5**, **6**, **7**, **8** and **10**, as well as of ligands **L2** and **L3** (1 mg/mL) were prepared in 100% MeOH, whereas stock solutions of complexes **2** and **3** (1 mg/mL) were prepared in 5% DMSO in deionised water. Positive control (1 mg/mL neostigmine bromide; Sigma-Aldrich, St. Louis, MO, USA) was also prepared in 100% MeOH. The stock solutions of the potential inhibitors and the positive and negative controls were added to the wells, and progressively diluted in 100 mM potassium phosphate buffer (pH 7.4) to the final volume of 50 μL. Then, 100 μL acetylthiocholine chloride (1 mM) and 5,5′-dithiobis-2-nitrobenzoic acid (0.5 mM) in 100 mM potassium phosphate buffer (pH 7.4) were added into the microtiter plate wells. Three ChEs were used as the enzyme sources: electric eel AChE (eeAChE); human recombinant AChE (hrAChE), and horse serum BChE (hsBChE) (all Sigma-Aldrich, St. Louis, MO, USA). These were dissolved in the same buffer to the final concentration of 0.0075 U/mL. Finally, 50 μL of each ChE solution was added into the microtiter plate wells to start the reaction, which was followed spectrophotometrically at 405 nm at 25 °C over 5 min using a kinetic microplate reader (Dynex Technologies Inc., Chantilly, VA, USA). The blank reactions without the inhibitors were run with the appropriate dilutions of the solvents in which the tested compounds were initially diluted (100% MeOH or 5% aqueous DMSO), and the readings were corrected according to the appropriate blanks. Each measurement was repeated at least three times. To determine the inhibitory constants (*K_i_*), the kinetics were monitored using three different final substrate concentrations (0.125, 0.25, 0.5 mM). The data were analysed using the OriginPro software (OriginPro 2020, OriginLab Corporation, Northampton, MA, USA).

#### 2.3.2. Glutathione S-Transferase Inhibition Assay

The activities of the GSTs were determined according to the method described by Habig et al. (1974) [48] using a cell imaging multi-mode reader (Cytation 3; BioTek, Winooski, VT, USA). The stock solutions of inhibitors were prepared as described for the ChE inhibition assays. Then the stock solutions of the potential inhibitors and negative controls were added to the wells, and progressively diluted in 100 mM sodium phosphate buffer (pH 6.5) to the final volume of 50 μL. 1-Chloro-2,4-dinitrobenzene (Sigma-Aldrich, St. Louis, MO, USA) was dissolved in ethanol to 50 mM, and then diluted with 100 mM sodium phosphate buffer (pH 6.5) to a final concentration of 4 mM. This solution (50 μL) and 2 mM reduced glutathione (100 μL) in the same buffer were added into the microtiter plate wells. Two GSTs were used as the enzyme sources: horse liver GST (hlGST) and human placenta GST (hGST) (Sigma-Aldrich, St. Louis, MO, USA). These were dissolved in 100 mM sodium phosphate buffer (pH 6.5), and 50 μL of these enzyme solutions were added into the wells to start the reaction. The final enzyme concentration was 0.044 U/mL. The blank reactions without the inhibitors were run with the appropriate dilutions of the solvents in which the tested compounds were initially diluted (100% MeOH or 5% aqueous DMSO), and the readings were corrected according to the appropriate blanks. The reactions were followed spectrophotometrically at 340 nm at 25 °C over 4 min. Each measurement was repeated at least three times. For determination of the inhibitory constants (*K_i_*), the kinetics were monitored using three different final substrate concentrations (200, 400, 800 μM). The data were analysed using the OriginPro software (OriginPro 2020, OriginLab Corporation, Northampton, MA, USA).

## 3. Results and Discussion

### 3.1. Tested Compounds and Synthesis of Organoruthenium Complexes

Ligands **L2**–**L3**, precursor **10** and synthesised complexes **2**–**8** (Figure 2) were evaluated for their inhibitory activities towards eeAChE, hrAChE, hsBChE, hlGST and hGST. Newly prepared complexes **2**–**5** and **7**–**8** were physiochemically characterised using ^1^H NMR, IR spectroscopy, elemental analysis (C, H, N), UV-Vis spectroscopy and HRMS. The ligands **L1**–**L3** and ruthenium precursors **9**–**10** used are commercially available. The synthesis, as well as physiochemical characterisation of complexes **1** and **6,** were previously reported in [8,33].

The organoruthenium(II) chlorido complex **1** with *O,S*-ligand pyrithione has been tested previously for its AChE, BChE and GST inhibition [12]. To further evaluate the influence of the monodentate halide ligands on biological activity of the ChEs and GSTs, the bromido **2** and iodido **3** analogues were prepared. Furthermore, to examine the influence of another type of bidentate ligand on the investigated system, the organoruthenium(II) chlorido complexes **4** and **5** with *O,O*-ligands were prepared following a modified procedure reported previously [45], using the chlorido ruthenium precursor **9** and the appropriate *β*-diketonate ligand **L2** or **L3**. Additionally, halide ligand Z was substituted by monodentate bulky phosphine pta ligand. Two organoruthenium(II) pta complexes with pyrithione ligand **L1** and *β*-diketonate ligand **L2** were prepared following a modified procedure published previously [30], to yield cationic complexes **6** and **7**, respectively. In order to also evaluate the activity of ruthenium complexes, derived from other ruthenium precursors than ruthenium precursor **9**, CORM complex **8** was synthesised.

### 3.2. Crystal Structures

Over the course of the study, new crystal structures of complexes **4** and **8** were obtained. Single crystals of complex **4** were prepared by liquid–liquid diffusion from a mixture of dichloromethane and hexane, and single crystals of complex **8** were prepared from a mixture of acetone and diethyl ether at room temperature. The crystallographic data and geometric parameters are given in Tables S1–S3.

**Figure 3:**
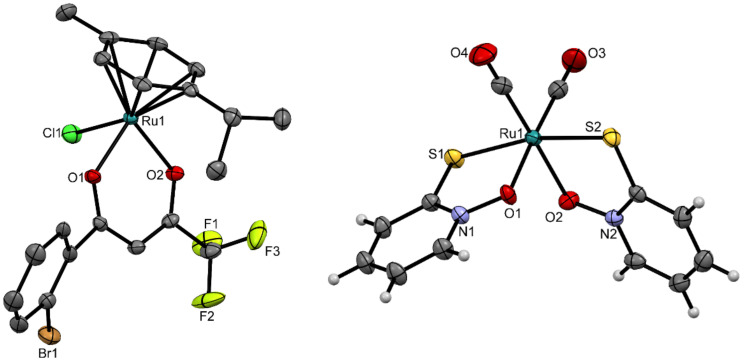
Crystal structures of complexes **4** (**left**) and **8** (**right**). Displacement ellipsoids are drawn at the 50% probability rate. Hydrogen atoms are omitted for clarity (**left**) or shown as spheres of arbitrary radii (**right**).

The ruthenium(II) chlorido complex **4** has a pseudo-octahedral “piano-stool” geometry, which is typical for organoruthenium(II) arene complexes with *O,O*-chelating ligands [49]. Crystal structure of complex **4** is shown in Figure 3. The ruthenium(II) ion was bound to the neutral *p*-cymene, chlorido ligand and a bidentate chelating *β*-diketonate ligand **L2**. The *η*^6^-arene ligand represents the “seat” of the piano stool, while the three remaining coordination sites have the roles of the “legs”. Complex **4** has bond lengths between the ruthenium(II) ion and the oxygen donor atoms of the *β*-diketonate ligand of 2.0810(19) Å and 2.0862(19) Å. A survey of the Cambridge Structural Database was performed for comparisons with known crystal structures. This included a number of ruthenium(II) compounds with *β*-diketonate ligands, and therefore only those compounds in which the *p*-cymene was coordinated to the ruthenium together with the *β*-diketonate ligand containing a –CF_3_ group were considered (structure codes: CUZZEE, CUZZII, KIMGAQ, KIMGEU, KIMGIY, KIMJEY, KIMJIC, KIMJOI, KIMLID, KIMLOJ, MIDNIX, NAYPEL, NAYPIP, NAYPOV, NAYPUB, WUNGIX, WUNGOD, WUNGUJ, WUNHAQ, WUNHEU, WUNHIY). The ruthenium-to-oxygen bond lengths ranged from 2.066 Å to 2.111 Å [50]. The bond lengths defined in the present study fit very well into the middle of this range.

The ruthenium pyrithione complex **8** has an octahedral geometry. Crystal structure of complex **8** is shown in Figure 3. The six-numbered coordination sphere of ruthenium(II) consists of two bidentate chelating pyrithione (**L1**) ligands, bound via the sulphur and oxygen donor atoms, and two neutral monodentate carbonyl ligands, bound via carbon atoms. The sulphur atoms from deprotonated pyrithione are positioned *trans* to each other. Only a few compounds were reported where pyrithione or its analogues were bound to ruthenium, and most of these were synthesised by the Turel research group. Pyrithione is usually bound in a deprotonated form in a bidentate manner via both oxygen and sulphur donor atoms. Therefore, only these structures were used for comparisons (structure codes: TOXVEK, TOXVIO, TOXVOU, TOXVUA, TOXWAH, TOXWEL, TOXWIP, TOXWOV, UQUZUD, URABAS). The bonds between ruthenium(II) and sulphur in complex **8** had lengths of 2.3711(6) Å and 2.3598(6) Å, which agrees well with the range from 2.334 Å to 2.370 Å in the structures from the Cambridge Structural Database [50]. In complex **8**, the distances between ruthenium and oxygen were 2.0859(16) Å and 2.1023(16) Å, which are also comparable to the lengths in the pyrithione-type organoruthenium(II) complexes in the Cambridge Structural Database [50].

### 3.3. Inhibition of Cholinesterases and GSTs by the Ruthenium-Based Complexes

In the present study, six organoruthenium(II) arene complexes with *β*-diketonate-type (**4**, **5**, **7**) or pyrithione-type ligands (**2**–**3** and **6**) were newly tested, along with the ruthenium precursor **10** and its new complex with pyrithione **8** towards eeAChE, hrAChE, hsBChE, hlGST and hGST. The compounds were first screened for the IC_50_ determination, and for those with IC_50_ < 33 μM, the inhibitory constants (*K_i_*) were determined. This threshold was chosen since currently approved and used anticholinesterase drugs exert their activity mostly in the low micromolar and in submicromolar range [51]. This paper also includes our previously published results of complex **1** and precursor **9** towards these enzymes [12]. The main objective of this study was to investigate the significance of the various structural elements of the newly prepared library of various organoruthenium complexes on the activities of selected ChEs as potential therapeutic drug targets that are involved in the pathogenesis of Alzheimer’s disease, and thus to further expand our previous data on ruthenium compounds with interesting activities [12,34]. Therefore, complexes were prepared from the ruthenium precursors **9** and **10** with various bidentate ligands (i.e., pyrithione **L1**, *β*-diketonates **L2**–**L3**) with different steric/electronic properties (i.e., different substituents on *β*-diketonates) together with various monodentate Z ligands (i.e., Cl^–^, Br^–^, I^–^, pta). Through this fine-tuning, insight could be gained into which structural elements are essential for the inhibition of the ChEs to determine the structure-activity relationship, and consequently to plan further synthesis optimisation. In addition, we investigated the possible inhibitory effects of these complexes on GST activities, as GSTs have essential roles in the development of anticancer drug resistance [25,26].

The discovery of new compounds that can simultaneously inhibit ChEs and GSTs would be interesting for treatment of patients who suffer from both Alzheimer’s disease and certain cancers, although some studies have suggested mutual exclusion of these two diseases in the same patient [52]. Tested ruthenium complexes have shown various activities on ChEs and GSTs described below, whereas all three ligands, i.e., **L1** [12], **L2** and **L3** showed no activities against these ChEs and GSTs.

Among the prepared library of compounds, the organoruthenium(II) pyrithione complexes **2** and **3** with bromide and iodide ligands, respectively, showed inhibitory activities in the low micromolar range against all of the ChEs and GSTs tested (i.e., eeAChE, hrAChE, hsBChE, hlGST, hGST). Instead, the organoruthenium(II) chlorido complexes with the *β*-diketonate ligands, i.e., complexes **4** and **5**, selectively inhibited only hsBChE and hlGSTs in the pharmacologically relevant micromolar range. Interestingly, the organoruthenium(II) pta complex **6** with pyrithione and the organoruthenium(II) pta complex **7** with *β*-diketonate ligand selectively inhibited only hsBChE, and the CORM complex **8** with pyrithione selectively inhibited only GSTs. The inhibition parameters for these compounds against ChEs and GSTs (i.e., IC_50_, *K_i_*) are shown in Table 1, Table 2, Table 3.

Complexes **2** and **3** are bromido and iodido analogues of the chlorido complex **1**, which was first described by Ristovski et al. [12]. The inhibitory activities of the bromido and iodido analogues against the ChEs were in the low micromolar range (Table 1) with IC_50_ values of, respectively, 13.14 μM and 12.90 μM against eeAChE, 6.57 μM and 6.55 μM against hrAChE, and 3.39 μM and 3.48 μM against hsBChE. These data are comparable to those obtained with the parent chlorido complex **1** [12] for eeAChE, hsBChE and hlGST. However, there were some important differences that can be highlighted, such as the higher susceptibility of hrAChE for these compounds compared to complex **1**, and the higher susceptibility of BChE over AChE. The higher susceptibility of hrAChE to the Br^–^/I^–^ analogues in comparison to the Cl^–^ analogue might be due to the lower sensitivity of the hrAChE used in the earlier study [12], where the structural analysis showed that half of the molecules in the crystal were blocked by the peptide loop that was formed by amino acid residues 483–491 [53]. The repeated testing of the inhibitory potential of complex **1** towards the hrAChE lot used in the present study confirmed that the observed differences were due to an enzyme structural defect, and not to the different monodentate Z ligands (i.e., Cl^–^, Br^–^, I^–^, pta). Both the Br^–^ and I^–^ analogues inhibited the ChEs in a pharmaceutically interesting low micromolar range [51] and were comparable to other ruthenium-based cholinesterase inhibitors, with IC_50_ values reported in the range of 0.2 μM to 50 μM [11,54,55,56,57]. Inhibition of all three ChEs was reversible and competitive, with *K_i_* values in the low micromolar range (Figures S1–S2). Considering the type of inhibition, we can conclude that these inhibitors most likely interact with the active site within the enzyme gorge. Further, the Br^–^ and I^–^ analogues also showed effective inhibition of the two GSTs, with IC_50_ values, respectively, of 3.39 μM and 3.07 μM for hlGST, and 4.64 μM and 15.97 μM for hGST. Considering the inhibition of hlGST, these data are similar to those obtained for complex **1**. On the other hand, compared with the I^–^ analogue **3** and complex **1**, the IC_50_ values for the Br^–^ analogue **2** against hGST were lower by factors of about 3 and 10, respectively, which indicated the importance of the monodentate Z ligand for the inhibitory activity. The inhibition was again reversible and competitive for both compounds tested against both GSTs (Figures S1 and S2).

Compared with the organoruthenium(II) pyrithione complex with the chlorido ligand, as complex **1** [12], complexes **2** and **3** showed slightly lower inhibitory potential against eeAChE, but comparable inhibitory activity against hlGST, and even better inhibitory activities against hrAChE, hsBChE and hGST. However, it is not disputed that changes in Z ligands (e.g., Br^–^, I^–^ instead of Cl^–^) play major roles in increasing inhibitory potential of organoruthenium(II) pyrithione complexes **2** and **3** with bromido or iodido ligands, respectively, against hsBChE and hGST. This effect is particularly striking against hGST, where the bromido ligand of complex **2** promoted an IC_50_ lower by approximately a factor of 10 compared to the chlorido ligand of complex **1**. The differences in inhibition of complexes **1**–**3** might be partly a consequence of various hydrolysis rates of the monofunctional halido leaving groups, but might also be related to changes in hydrophobicity, as well as solubility. Importantly, the hydrolysis rates of halido ions are reported to be connected to the activation of the complexes, as the substitution of the negatively charged halido ligands with neutral water ligand results in positively charged metal species that can further interact with biological targets via electrostatic interactions [9,58,59,60].

In addition, this study also investigated the inhibitory activity of organoruthenium(II) pyrithione complex **6** with the pta ligand, which effectively inhibited only hsBChE with IC_50_ value of 0.5 μM. The IC_50_ value of complex **6** is lower by approximately a factor of 15 compared to complexes **1** and **2**, and a factor 7 for complex **3**. This compound compared to previously reported pyrithione compounds (Cl^–^, Br^–^, I^–^) shows us that the changes in the Z ligands might result in alterations of the inhibitory activities or in alterations to the specificities towards the enzymes used in the present study. As a selective BChE inhibitor, this compound could be of interest for further preclinical studies; however, its activity should be tested also on BChE of a human origin that was not commercially available during the course of this study.

The combined results here thus demonstrate the importance of a suitable Z ligand choice in such organoruthenium(II) pyrithione complexes for the fine tuning of their inhibitory potentials against enzymes of human and other animal origins.

Other compounds studied here were the organoruthenium(II) complexes in which the bidentate ligands were *β*-diketonates with various substituents and the monodentate Z ligands Cl^–^ (complexes **4** and **5**) or pta (complex **7**). The data given in Table 2 show that all of these compounds effectively inhibited hsBChE in low micromolar range, with IC_50_ values of 30.98 μM for complex **4**, 31.99 μM for complex **5**, and 19.2 μM for complex **7**. The IC_50_ values of complexes **4**, **5**, and **7** are about 2.5 to 9 times higher compared to the IC_50_ values of complexes **1** and **2** or **3**, respectively. Moreover, complex **7** expressed selective inhibitory activity towards animal BuChE. On the other hand, the chlorido compounds **4** and **5** also inhibited hlGST with IC_50_ values of 16.11 μM and 18.28 μM, respectively. However, complex **4** did not inhibit hGST in the concentration range of interest, and another compound, complex **5**, did not inhibit hGST at all. In all of these cases, the inhibition was reversible and competitive, with *K_i_* values in the low micromolar range (Figure S3).

Obtained results show that the different substituents of *β*-diketonates in the organoruthenium(II) cholorido complexes **4** and **5** had no effects on the inhibitory activities or on the specificities against the ChEs and GSTs used in the present study. This was demonstrated by using different substituents on the *β*-diketonates with the same Z ligand as Cl^–^, where neither the inhibitory activity nor the avidity towards the different ChEs and GSTs changed. Both of these compounds inhibited hsBChE and hlGST with very similar IC_50_ values.

Replacement of the Z ligand Cl^–^ with pta, as complex **7**, resulted in a slight improvement of the inhibitory activity against hsBChE, but led to the loss of susceptibility against hlGST, and also to the other enzymes tested. This indicates that the nature of the Z ligand in the organoruthenium(II) *β*-diketonate complexes affects the inhibitory activities of the respective compounds on these ChEs and GSTs.

Nowadays, the development of safe and efficient CORMs as therapies for neurovascular diseases is very important [61]. In the central nervous system, a protective role of low-concentration dose CO has been reported, which has suggested beneficial effects in diseases such as Alzheimer’s disease, traumatic brain injury and stroke [61,62]. Should a compound simultaneously inhibit ChEs and release CO, it might have a dual beneficial effect in the treatment of Alzheimer’s disease. In the presented study, we investigated inhibition of tested enzymes by ruthenium precursor **10** and its complex with pyrithione **8** to evaluate the influence of the chosen metal precursor. Complex **8** was prepared from the ruthenium CORM precursor **10**. The pyrithione ligand was chosen instead of the *β*-diketonate ligand because pyrithione complexes have generally shown better inhibition of ChEs and GSTs The ruthenium precursor **10** and its complex with pyrithione **8** were also included in the study to evaluate the influence of the chosen metal precursor. The pyrithione ligand was chosen instead of the *β*-diketonate ligand because pyrithione complexes have generally shown better inhibition of ChEs and GSTs. As shown by the data given in Table 3, complex **10** efficiently inhibited hlGST (IC_50_ = 9.76 μM) and hGST only weakly, but did not have any effects on the ChEs which showed activity in the pharmaceutically interesting range only for hlGST inhibition (IC_50_ = 9.76 μM). Meanwhile, unlike the precursor **9**, when pyrithione was complexed with **10** to obtain complex **8**, this showed effective inhibition of the GSTs of both animal and human origins, with IC_50_ values of 3.66 μM for hlGST and 16.61 μM for hGST. These data show that complex **8** selectively inhibits GSTs, which makes it interesting for further preclinical studies. However, complex **8** showed no activity against ChEs. The inhibition of both of these GSTs was again reversible and competitive (Figure S4). Future preclinical studies in cells and mammalian organisms would be necessary to confirm that these complexes selectively inhibit the enzymes of interest and can be considered as potential anticholinesterase and anti-GST drugs.

## 4. Conclusions

A small library of five novel organoruthenium(II) compounds with *p*-cymene as an arene ligand was synthesised, along with one organoruthenium(II) carbonyl complex CORM, and crystal structures of complexes **4** and **8** were determined. Further, ligands **L1**–**L3**, precursor **10** and complexes **2**–**8** were screened for inhibitory activities against AChEs, BChEs and GSTs of human and other animal origins. The arene-organoruthenium(II) pyrithione complexes with Br^–^ (i.e., complex **2**) and I^–^ (i.e., complex **3**) as monodentate ligands inhibited all of these ChEs and GSTs at low micromolar concentrations, with no selectivity observed. Furthermore, the organoruthenium(II) *β*-diketonate complexes **4** and **5** that contain Cl^–^ inhibited hsBChE and hlGST, while pta complexes **6** and **7** selectively inhibited hsBChE in the low micromolar range. These data confirm that the organoruthenium(II) carbonyl complex with pyrithione (i.e., complex **8**) is a selective GST inhibitor, without ChE inhibitory activity. These data also demonstrate the importance of the nature of the ligands in the structure of these organoruthenium(II) complexes for their inhibitory activities against ChEs and GSTs, and they provide some interesting compounds for further preclinical testing as ChE and GST inhibitory drugs.

## Figures and Tables

**Table 1:** Inhibition of electric eel (eeAChE) and human recombinant acetylcholinesterase (hrAChE) by the ruthenium compounds **1**–**10** and the free ligands **L1**–**L3**.

		Enzyme Inhibition (μM)			
		eeAChE		hrAChE	
Compound	Code	IC_50_	*K*_i_	IC_50_	*K*_i_
[RuCym(L1)Cl] ^a^	**1**	5.01 ± 0.8	35.0	25.06 ± 2.5	24.0
[RuCym(L1)Br]	**2**	13.14 ± 2.5	4.87	6.57 ± 4.1	2.49
[RuCym(L1)I]	**3**	12.90 ± 2.0	1.43	6.55 ± 4.5	1.94
[RuCym(L2)Cl]	**4**	/	/	/	/
[RuCym(L3)Cl]	**5**	/	/	/	/
[RuCym(L1)pta]PF_6_ ^b^	**6**	/	/	/	/
[RuCym(L2)pta]PF_6_	**7**	/	/	/	/
[Ru(L1)_2_(CO)_2_]	**8**	/	/	/	/
[Ru(*p*-cymene)Cl_2_]_2_ ^a^	**9**	>100	/	>100	/
[Ru(CO)_3_Cl_2_]_2_	**10**	>100	/	/	/
1-Hydroxypyridine-2(1*H*)-thione	**L1**	>100	/	/	/
1-(2-Bromophenyl)-4,4,4-trifluorobutane-1,3-dione	**L2**	/	/	/	/
1-Phenylicosane-1,3-dione	**L3**	/	/	/	/
Neostigmine methylsulphate		5.98 ± 1.0	/	/	/

^a^, Inhibition of eeAChE and hrAChE by **1** and **9** was previously reported in [12]; ^b^, The synthesis of the complex **6** was previously published in [33]; IC_50_, Concentration required to induce 50% inhibition of enzyme activity; *K_i_*, Inhibition constants determined for compounds with IC_50_ < 33 μM. Data are means ± SEM of three independent measurements; /, No activity.

**Table 2:** Inhibition of horse serum butyrylcholinestarse (hsBChE) by the ruthenium compounds **1**–**10** and the free ligands **L1**–**L3**.

		Enzyme Inhibition (μM)	
		hsBChE	
Compound	Code	IC_50_	*K*_i_
[RuCym(L1)Cl] ^a^	**1**	7.52 ± 1.3	4.0
[RuCym(L1)Br]	**2**	3.39 ± 2.3	0.63
[RuCym(L1)I]	**3**	3.48 ± 0.9	0.80
[RuCym(L2)Cl]	**4**	30.98 ± 2.1	6.19
[RuCym(L3)Cl]	**5**	31.99 ± 2.5	8.84
[RuCym(L1)pta]PF_6_ ^b^	**6**	0.39 ± 0.79	1.1
[RuCym(L2)pta]PF_6_	**7**	19.20 ± 1.5	9.26
[Ru(L1)_2_(CO)_2_]	**8**	/	/
[Ru(*p*-cymene)Cl_2_]_2_ ^a^	**9**	32.70 ± 4.3	/
[Ru(CO)_3_Cl_2_]_2_	**10**	>100	/
1-Hydroxypyridine-2(1*H*)-thione	**L1**	>100	/
1-(2-Bromophenyl)-4,4,4-trifluorobutane-1,3-dione	**L2**	/	/
1-Phenylicosane-1,3-dione	**L3**	/	/
Neostigmine methylsulphate		92.70 ± 2.2	/

^a^, Inhibition of hsBChE by **1** and **9** was previously reported in [12]; ^b^, The synthesis of the complex **6** was previously published in [33]; IC_50_, Concentration required to induce 50% inhibition of enzyme activity; *K_i_*, Inhibition constants determined for compounds with IC_50_ < 33 μM. Data are means ± SEM of three independent measurements; /, No activity.

**Table 3:** Inhibition of horse liver (hlGST) and human placenta glutathione S-transferase (hGST) by the ruthenium compounds **1**–**10** and the free ligands **L1**–**L3**.

		Enzyme Inhibition (μM)			
		hlGST		hGST	
Compound	Code	IC_50_	*K*_i_	IC_50_	*K*_i_
[RuCym(L1)Cl] ^a^	**1**	2.26 ± 0.5	10.0	45.0 ± 5.2	/
[RuCym(L1)Br]	**2**	<3.39	0.79	4.64 ± 3.7	4.08
[RuCym(L1)I]	**3**	<3.07	1.60	15.97 ± 3.0	8.60
[RuCym(L2)Cl]	**4**	16.11 ± 6.5	2.83	>100	/
[RuCym(L3)Cl]	**5**	18.28 ± 5.3	1.83	/	/
[RuCym(L1)pta]PF_6_ ^b^	**6**	/	/	*	*
[RuCym(L2)pta]PF_6_	**7**	/	/	/	/
[Ru(L1)_2_(CO)_2_]	**8**	<3.66	0.85	16.61 ± 1.4	9.65
[Ru(*p*-cymene)Cl_2_]_2_ ^a^	**9**	>100	/	>100	/
[Ru(CO)_3_Cl_2_]_2_	**10**	9.76 ± 0.4	2.93	97.65 ± 1.0	/
1-Hydroxypyridine-2(1*H*)-thione	**L1**	/	/	/	/
1-(2-Bromophenyl)-4,4,4-trifluorobutane-1,3-dione	**L2**	/	/	/	/
1-Phenylicosane-1,3-dione	**L3**	/	/	/	/
Neostigmine methylsulphate		/	/	/	/

^a^, Inhibition of hlGST and hGST by **1** and **9** was previously reported in [12]; ^b^, The synthesis of the complex **6** was previously published in [33]; *, Compound was not tested; IC_50_, Concentration required to induce 50% inhibition of enzyme activity; *K_i_*, Inhibition constants determined for compounds with IC_50_ < 33 μM. Data are means ± SEM of three independent measurements; /, No activity.

## Data Availability

All of the data generated or analysed during this study are included in this published article (and its Supplementary Information files).

## Supplementary Materials

The following are available online at https://www.mdpi.com/article/10.3390/biomedicines9091243/s1, Figure S1: Dixon plots for determination of type of inhibition and inhibition constants (*K_i_*) for compound **2** against electric eel acetylcholinesterase (eeAChE), human recombinant acetylcholinesterase (hrAChE), horse serum butyrylcholinesterase (hsBChE), horse liver glutathione S-transferase (hlGST) and human placenta glutathione S-transferase (hGST). Substrate concentrations: acetylthiocholine (eeAChE, hrAChE, hsBChE), 0.125 mM (▲), 0.25 mM (●), 0.5 mM (■); 1-chloro-2,4-dinitrobenzene (hlGST, hGST), 200 μM (▲), 400 μM (●), 800 μM (■), Figure S2: Dixon plots for determination of type of inhibition and inhibition constants (*K_i_*) for compound **3** against electric eel acetylcholinesterase (eeAChE), human recombinant acetylcholinesterase (hrAChE), horse serum butyrylcholinesterase (hsBChE), horse liver glutathione S-transferase (hlGST) and human placenta glutathione S-transferase (hGST). Substrate concentrations: acetylthiocholine (eeAChE, hrAChE, hsBChE), 0.125 mM (▲), 0.25 mM (●), 0.5 mM (■); 1-chloro-2,4-dinitrobenzene (hlGST, hGST), 200 μM (▲), 400 μM (●), 800 μM (■), Figure S3: Dixon plots for determination of type of inhibition and inhibition constants (*K_i_*) for compounds **4**, **5** and **7** against horse serum butyrylcholinesterase (hsBChE) and horse liver glutathione S-transferase (hlGST). Substrate concentrations: acetylthiocholine (hsBChE), 0.125 mM (▲), 0.25 mM (●), 0.5 mM (■); 1-chloro-2,4-dinitrobenzene (hlGST), 200 μM (▲), 400 μM (●), 800 μM (■), Figure S4: Dixon plots for determination of type of inhibition and inhibition constants (*K_i_*) for precursor **10** and compound **8** against horse liver glutathione S-transferase (hlGST). Substrate concentrations: 1-chloro-2,4-dinitrobenzene, 200 μM (▲), 400 μM (●), 800 μM (■), Table S1: Crystallographic data for compounds **4** and **8**, Table S2: Relevant bond lengths and angles in compound **4**, Table S3: Relevant bond lengths and angles in compound **8**.
