# Supplementary material for: Fine Tuning of Cholinesterase and Glutathione-S-Transferase Activities by Organoruthenium(II) Complexes
Source: Biomedicines. 2021 Sep 16;9(9):1243. doi: 10.3390/biomedicines9091243 (PMC8467340; doi:10.3390/biomedicines9091243)
Supplement: Supplementary file 1 [file biomedicines-09-01243-s001.zip › biomedicines-1316377-supplementary.pdf]

Table S1. Crystallographic data for compounds **4** and **8**.

Parameter	Compound	
	**4**	**8**
Empirical formula	$C_{20}H_{19}BrClF_3O_2Ru$	$C_{12}H_8N_2O_4RuS_2$
Formula weight	564.78	409.39
Crystal system	orthorhombic	triclinic
Space group	*P b c a*	*P* –1
T [K]	150.00(10)	150.05(10)
λ [Å]	0.71073	0.71073
a [Å]	9.1573(3)	7.4914(4)
b [Å]	20.5173(7)	9.8535(6)
c [Å]	21.4528(7)	10.1047(5)
α [°]	90	85.849(4)
β [°]	90	74.188(4)
γ [°]	90	85.560(4)
V [Å^3]	4030.6(2)	714.49(7)
Z	8	2
D_{calc} [g/cm^3]	1.861	1.903
μ [mm^{-1}]	2.933	1.404
F(000)	2224	404
Size [mm^3]	0.5×0.3×0.08	0.2×0.15×0.04
Collected reflections	20571	7311
Unique reflections	5525	3741
Observed reflections	4384	3195
R_{int}	0.0377	0.0302
R_1 ($I > 2\sigma(I)$)	0.0350	0.0281
wR_2 ($I > 2\sigma(I)$)	0.0763	0.0568
R_1 (all data)	0.0525	0.0378
wR_2 (all data)	0.0850	0.0602

Table S2. Relevant bond lengths and angles in compound **4**.

**Bonds [Å]**	
Ru1–Cl1	2.4024(8)
Ru1–O1	2.0810(19)
Ru1–O2	2.0862(19)
Ru1–C21	2.193(3)
Ru1–C22	2.176(3)
Ru1–C23	2.158(3)
Ru1–C24	2.201(3)
Ru1–C25	2.159(3)
Ru1–C26	2.164(3)
**Angles [°]**	
O1–Ru1–Cl1	84.31(6)
O1–Ru1–O2	87.19(7)
O1–Ru1–C21	126.59(10)
O1–Ru1–C22	163.73(10)
O1–Ru1–C23	145.27(10)
O1–Ru1–C24	108.32(9)
O1–Ru1–C25	87.96(9)
O1–Ru1–C26	95.83(9)
O2–Ru1–Cl1	84.88(6)
O2–Ru1–C21	145.15(10)
O2–Ru1–C22	108.97(10)
O2–Ru1–C23	88.51(10)
O2–Ru1–C24	96.38(9)
O2–Ru1–C25	127.64(10)
O2–Ru1–C26	165.18(10)
C21–Ru1–Cl1	89.85(8)
C21–Ru1–C24	81.92(11)
C22–Ru1–Cl1	98.68(9)
C22–Ru1–C21	37.95(11)
C22–Ru1–C24	68.95(11)
C23–Ru1–Cl1	129.56(8)
C23–Ru1–C21	68.66(12)
C23–Ru1–C22	37.92(11)
C23–Ru1–C24	38.15(11)
C23–Ru1–C25	67.88(11)
C23–Ru1–C26	80.73(11)
C24–Ru1–Cl1	167.34(8)
C25–Ru1–Cl1	146.17(8)
C25–Ru1–C21	68.77(11)
C25–Ru1–C22	80.66(11)
C25–Ru1–C24	37.55(11)
C25–Ru1–C26	38.36(11)
C26–Ru1–Cl1	109.83(9)
C26–Ru1–C21	37.72(11)
C26–Ru1–C22	68.06(11)
C26–Ru1–C24	68.88(11)

Table S3. Relevant bond lengths and angles in compound **8**.

**Bonds [Å]**	
Ru1–S1	2.3711(6)
Ru1–S2	2.3598(6)
Ru1–O1	2.0859(16)
Ru1–O2	2.1023(16)
Ru1–C30	1.860(3)
Ru1–C40	1.856(3)
**Angles [°]**	
S2–Ru1–S1	167.08(2)
O1–Ru1–S1	83.32(4)
O1–Ru1–S2	85.76(4)
O1–Ru1–O2	84.84(6)
O2–Ru1–S1	88.71(5)
O2–Ru1–S2	83.43(5)
C30–Ru1–S1	97.52(8)
C30–Ru1–S2	93.08(8)
C30–Ru1–O1	177.26(9)
C30–Ru1–O2	92.56(9)
C40–Ru1–S1	92.17(8)
C40–Ru1–S2	94.99(8)
C40–Ru1–O1	91.37(9)
C40–Ru1–O2	175.98(9)
C40–Ru1–C30	91.21(11)

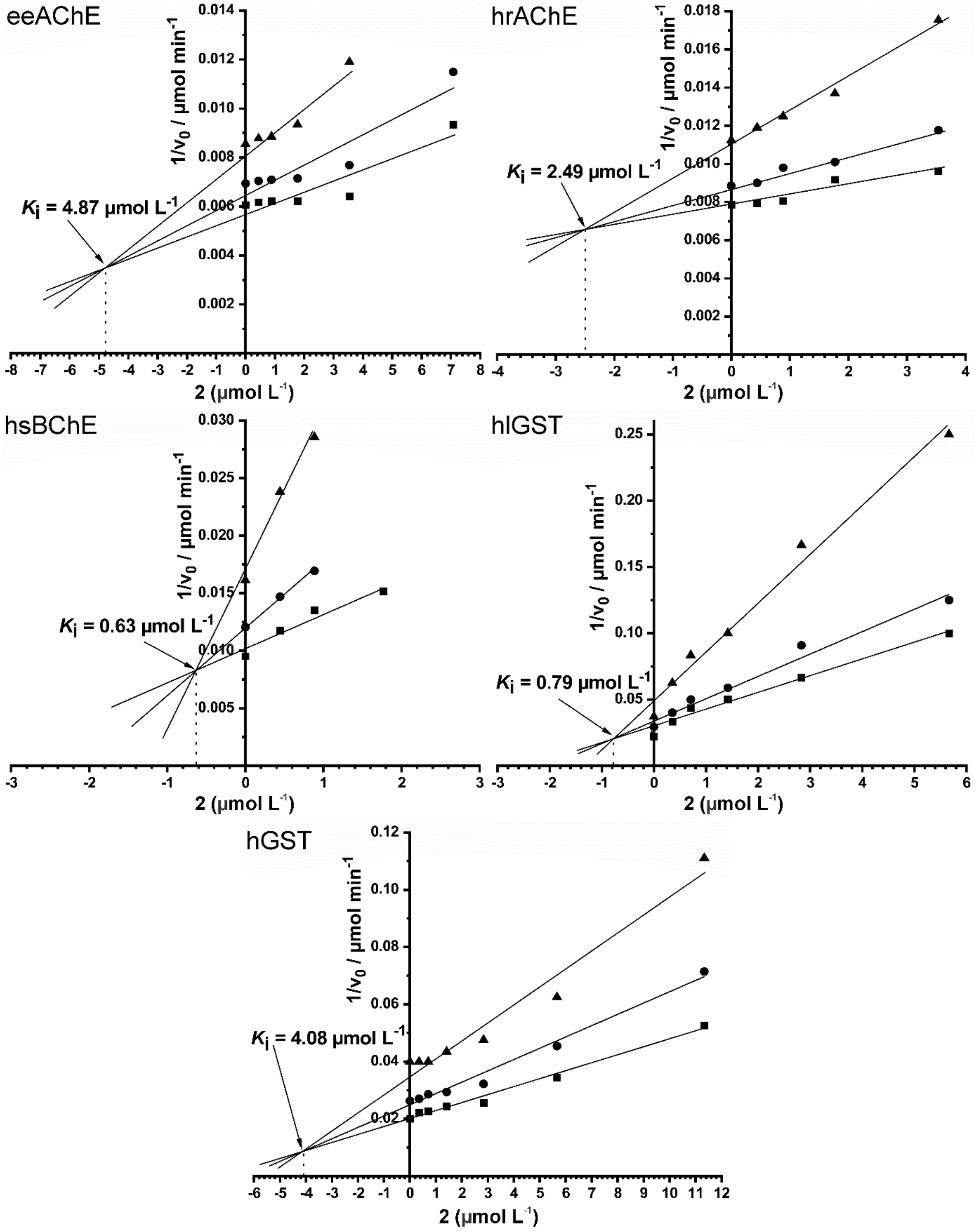

Figure S1. Dixon plots for determination of type of inhibition and inhibition constants (K_i) for compound **2** against electric eel acetylcholinesterase (eeAChE), human recombinant acetylcholinesterase (hrAChE), horse serum butyrylcholinesterase (hsBChE), horse liver glutathione S-transferase (hlGST) and human placenta glutathione S-transferase (hGST). Substrate concentrations: acetylthiocholine (eeAChE, hrAChE, hsBChE), 0.125 mM (▲), 0.25 mM (●), 0.5 mM (■); 1-chloro-2,4-dinitrobenzene (hlGST, hGST), 200 µM (▲), 400 µM (●), 800 µM (■).

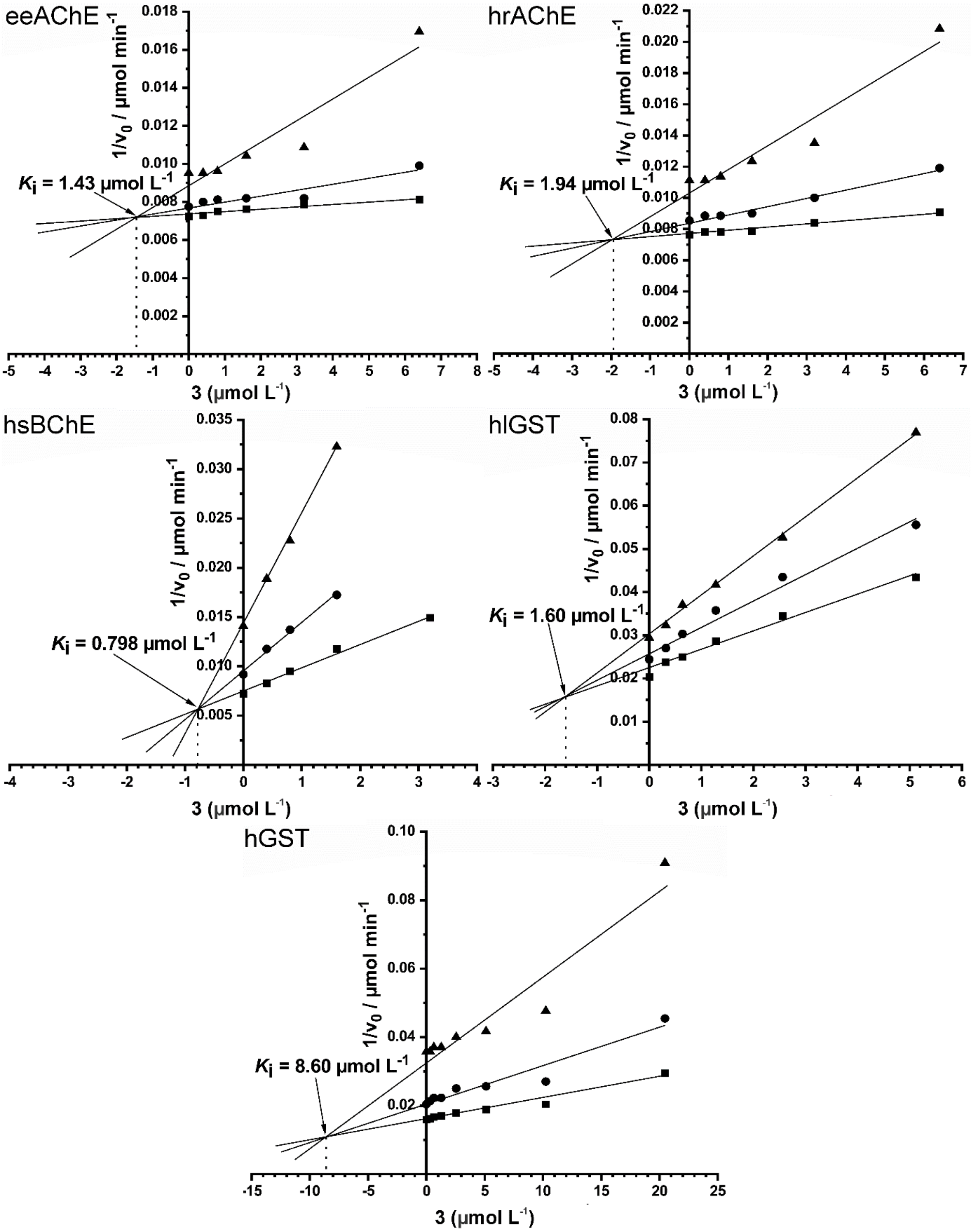

Figure S2. Dixon plots for determination of type of inhibition and inhibition constants (K_i) for compound **3** against electric eel acetylcholinesterase (eeAChE), human recombinant acetylcholinesterase (hrAChE), horse serum butyrylcholinesterase (hsBChE), horse liver glutathione S-transferase (hlGST) and human placenta glutathione S-transferase (hGST). Substrate concentrations: acetylthiocholine (eeAChE, hrAChE, hsBChE), 0.125 mM (▲), 0.25 mM (●), 0.5 mM (■); 1-chloro-2,4-dinitrobenzene (hlGST, hGST), 200 μM (▲), 400 μM (●), 800 μM (■).

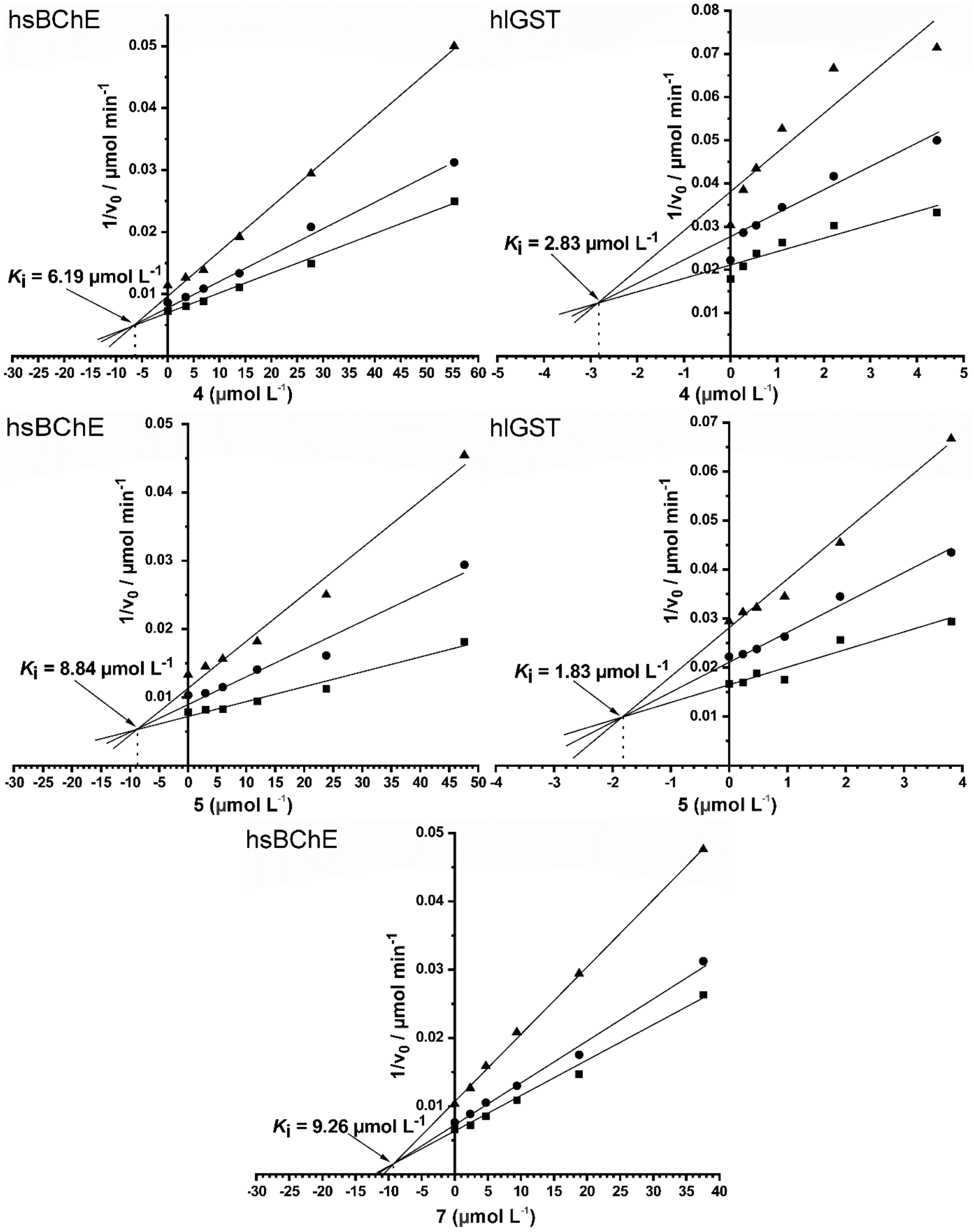

Figure S3. Dixon plots for determination of type of inhibition and inhibition constants (K_i) for compounds **4**, **5** and **7** against horse serum butyrylcholinesterase (hsBChE) and horse liver glutathione S-transferase (hlGST). Substrate concentrations: acetylthiocholine (hsBChE), 0.125 mM (▲), 0.25 mM (●), 0.5 mM (■); 1-chloro-2,4-dinitrobenzene (hlGST), 200 µM (▲), 400 µM (●), 800 µM (■).

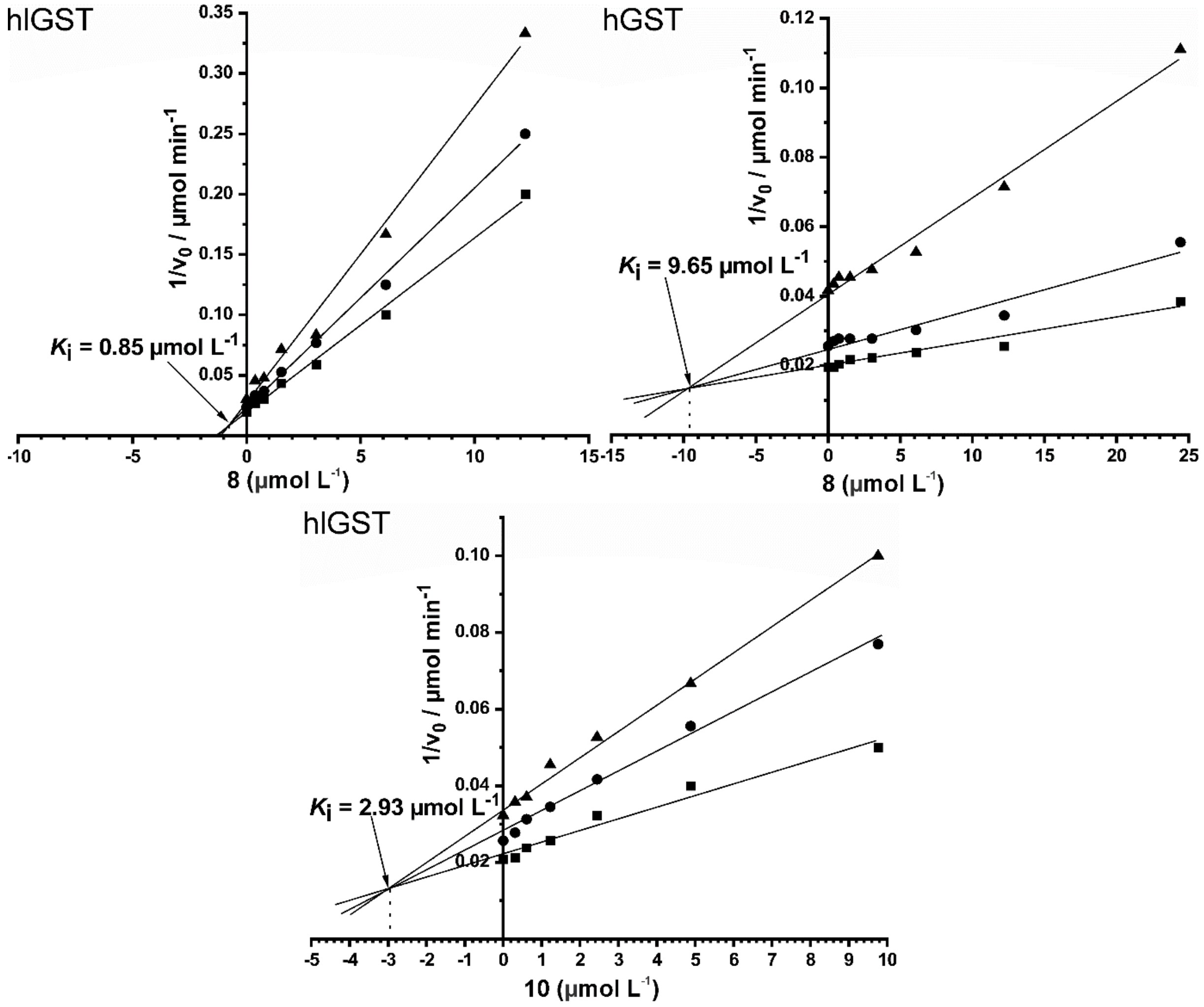

Figure S4. Dixon plots for determination of type of inhibition and inhibition constants (K_i) for precursor **10** and compound **8** against horse liver glutathione S-transferase (hlGST). Substrate concentrations: 1-chloro-2,4-dinitrobenzene, 200 µM (▲), 400 µM (●), 800 µM (■).
